# Host choice of *Phlebotomus orientalis* (Diptera: Psychodidae) in animal baited experiments: a field study in Tahtay Adiyabo district, northern Ethiopia

**DOI:** 10.1186/s13071-015-0807-4

**Published:** 2015-03-31

**Authors:** Araya Gebresilassie, Solomon Yared, Essayas Aklilu, Oscar David Kirstein, Aviad Moncaz, Habte Tekie, Meshesha Balkew, Alon Warburg, Asrat Hailu, Teshome Gebre-Michael

**Affiliations:** Department of Zoological Sciences, Addis Ababa University, Addis Ababa, Ethiopia; Department of Biology, College of Natural Science, Jigjiga University, Jigjiga, Ethiopia; Department of Microbiology and Molecular Genetics, The Institute of Medical Research Israel-Canada The Kuvin Center for the Study of Infectious and Tropical Diseases, Faculty of Medicine, The Hebrew University, Hadassah Medical School, Jerusalem, Israel; Aklilu Lemma Institute of Pathobiology, Addis Ababa University, Addis Ababa, Ethiopia; Department of Microbiology, Immunology and Parasitology, College of Health Sciences, Addis Ababa University, Addis Ababa, Ethiopia

**Keywords:** Host attractiveness, *Phlebotomus orientalis*, Visceral leishmaniasis, Zoophilic sand flies

## Abstract

**Background:**

Host choice and feeding success of sand fly vectors of visceral leishmaniasis (VL) are important factors in understanding the epidemiology and for developing efficient control strategies. The aim of the present study was to determine the host preference of *Phlebotomus orientalis* in the VL focus of Tahtay Adiyabo district, northern Ethiopia.

**Methods:**

Two separate experiments were conducted testing attraction of *P. orientalis* to humans, domestic animals, and small wild animals. The host choice of *P. orientalis* and other sand fly species was assessed using tent traps baited with seven different animals (human, cow, sheep, goat, donkey, dog and chicken) and a blank control. Baited traps were rotated every night in a Latin square design for two consecutive full rounds totaling 16 trap-nights. The second set of experiments tested attraction to small wild animals including; ground squirrel (*Xerus rutilus*), hare (*Lepus* sp.), gerbil (*Tatera robusta*) and spiny mouse (*Acomys cahirinus*). Animals were caged in standard rodent traps or cylindrical wire-mesh cages. The bait animals were placed in agricultural field and the attracted sand flies were collected using unlit CDC traps for 10 trapping nights. Sand fly specimens collected from each of the experiments were identified to species level and counted.

**Results:**

Significant difference (*P* < 0.05) was observed in the attraction and feeding rate of *P. orientalis* to different baits. In the first experiment, cow-baited tent traps attracted the highest mean number of *P. orientalis* (mean = 510 flies). The engorgement rate of *P. orientalis* on donkey was the highest followed by cow, and much lower on goat, sheep, dog and chicken. In the case of smaller wild animals, more numbers of *P. orientalis* females were attracted to squirrels followed by hares, gerbils and the spiny rat. However, the engorgement rates for *P. orientalis* in the smaller animals were very low (1.08%) compared with larger domestic animals (30.53%).

**Conclusion:**

The tendency of female *P. orientalis* to engorge in large numbers on certain species of domestic as well as wild animals strongly indicated that the species is primarily zoophilic in its host preference with feeding habits that may vary depending on the availability of hosts.

## Background

Ninety-eight countries and 3 territories on 5 continents are endemic for either of the two major forms of leishmaniasis: cutaneous leishmaniasis (CL), a disfiguring and stigmatizing disease, and visceral leishmaniasis (VL) or kala-azar, which is fatal if untreated [1,2]. In Ethiopia, VL caused by *Leishmania donovani*, is considered an emerging disease with an estimated incidence of 3,700 to 7,400 cases per year [2]. Highly VL endemic foci are in the south‐west and the Humera and Metema lowlands in the north‐west of the country [3,4]. However, recently increasing numbers of VL has been reported from previously non-endemic regions such as Libo Kemkem district of Amhara Regional State and Tahtay Adiyabo district in Tigray Regional State in Northern Ethiopia [5,6].

The various forms of leishmaniasis, including VL are transmitted by the bite of infected female sand flies of the genus *Phlebotomus* in the Old World and *Lutzomyia* in the New World [7,8]. Transmission of VL occurs when a sand fly acquires infection during feeding on an infected host and transmits the parasite during subsequent feeding after completion of the gonotrophic cycle, during which the parasite multiplies in the midgut and migrates to the foregut and mouthparts of the infected sand fly female. In southern Ethiopia and Kenya, the principal vector is *P. martini* which breeds and rests in termite mounds [9,10], whereas in northern Ethiopia and eastern Sudan, *P. orientalis* is implicated as the vector inhabiting *Acacia* forests and cracking vertisols [11-13].

The host preferences of several sand fly species have been investigated mostly through the identification of the sources of bloodmeals using serological [14,15] or molecular assays [16,17] and host attractiveness experiments [18-20]. Previously, the host preference of *P. orientalis* in Sudan and Ethiopia was determined by quantifying the host preferences using different animal baits [20,21] or by identification of sources of bloodmeals by ELISA [12]. In eastern Sudan, it has been shown that *P. orientalis* is largely attracted to dogs, which is a suspected reservoir host for domestic transmission of VL in the area [13]. In the Humera-Metema plains of northwest Ethiopia, the host preference of *P. orientalis* appeared to be zoophilic, predominantly feeding on bovine blood [13]. These limited studies in East Africa might indicate *P. orientalis* to be an opportunistic feeder; however, detailed studies are needed to understand the natural host preference profile of *P. orientalis* and other sand fly species and their possible epidemiological significance of both domestic and wild animals in the transmission dynamics of VL.

Taking into account the fragmentary information available on the feeding habit of *P. orientalis*, we designed an experiment to determine the relative host attractiveness and feeding success of *P. orientalis* on domestic and small wild mammals in a VL endemic area of north Ethiopia.

## Methods

### Study area

The study area has previously been described elsewhere [22]. Briefly, the investigation was conducted in the Geza Adura sub-village in the Tahtay Adiyabo district (14°22’27” N/37°44’36” E) in Tigray Regional State, Northern Ethiopia, which is situated 1,117 km north of Addis Ababa. The climate is generally sub-tropical-arid, with an extended dry period of nine to ten months (October-May), experiencing only one rainy season (June-September) with a mean annual precipitation of about 600 mm (Ethiopian National Meteorological Agency). April and May are the warmest months with an average temperature of 39°C and January is the coldest with an average temperature of 14.2°C.

In different villages of Tahtay Adiyabo district, large numbers of domestic animals including cattle, sheep, goats, donkeys, camels, dogs and chickens are kept. Moreover, a wide range of wild animals such as hares, ground squirrels, rodents, reptiles, white-tailed mongoose and foxes are either occasionally or commonly seen.

### Host choice experiments

The two independent host choice studies under field conditions were conducted between March and April 2013 in rotational experimental design in which wild sand flies were given the choice of different animal baits. The experiments were conducted in an open agricultural field, where there were no potential bloodmeal source animals (cattle, sheep, goats, donkeys, camels, dogs, chickens and other small wild animals) near the test traps for at least 400–450 meters.

### Experiment I

**Figure 1:**
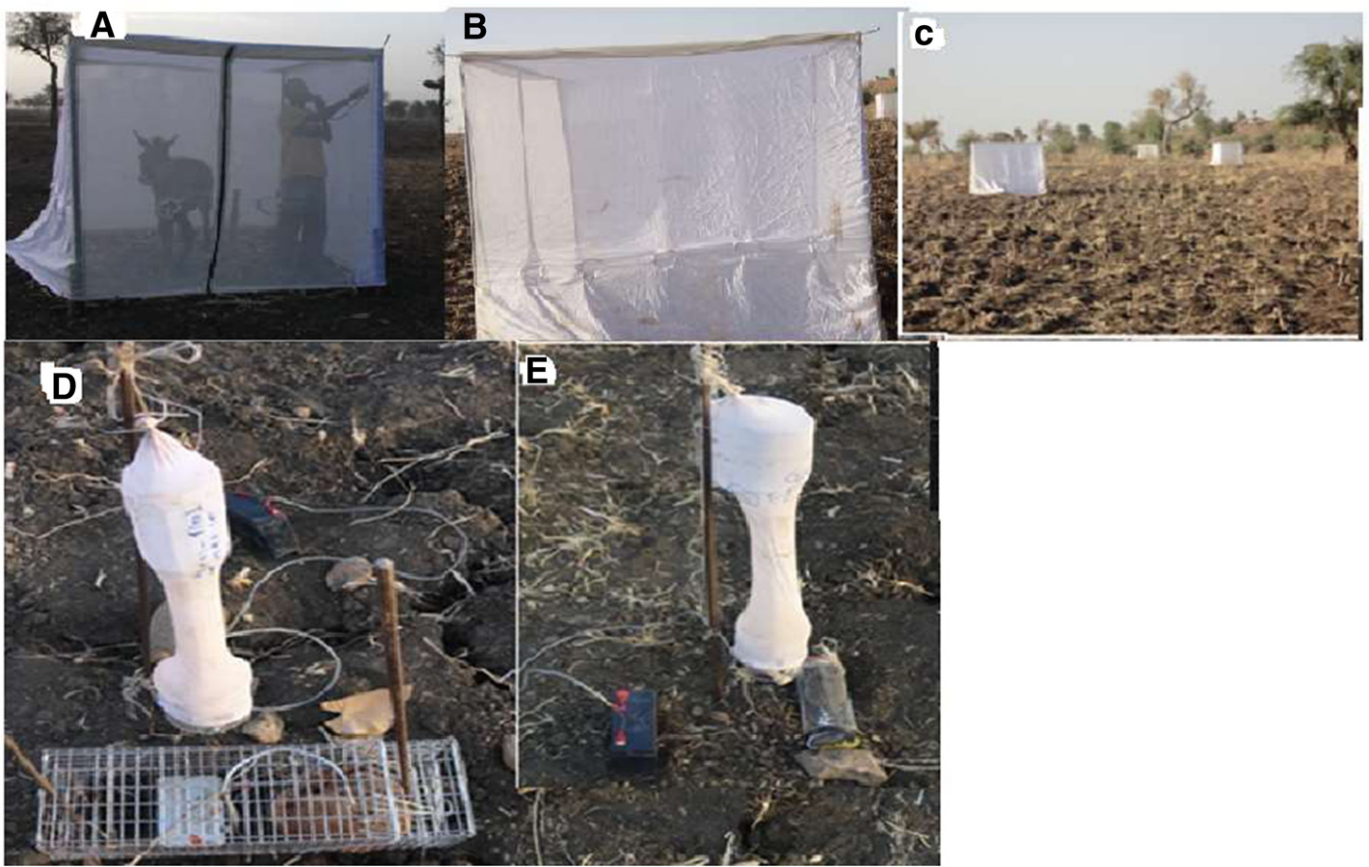
**Host attractiveness experiments. A**: Tent trap with animal bait. **B**: Cones fitted to one side of the tent. **C**: Arrangement of animal baits in the field. **D**: Cage trap baited with ground squirrel. **E**: Cage baited with rodent species.

The experiment was conducted using wild sand flies offered a choice of seven baits (human volunteer, cow, sheep, goat, donkey, dog and chicken) and control (without baits) assigned in tent traps. Each tent trap (dimension: 2.5 m × 2.5 m × 2.5 m) was constructed from transparent sand fly-proof netting supported by four rectangular metal frames and four metal poles to firmly fix them to the ground when installed (Figure 1-A). Six cone-shaped openings were fitted on the sides of the tent and the tent was raised a few centimeters above the ground to allow entry of host seeking sand flies (Figure 1-B). One side of the net had a long zipped slit (top to bottom) to enable entry and exit of host plus the participant to aspirate sand flies from inside the trap. The tent trap design was based on a prototype used previously to assess sand fly attraction behavior in Colombia [19].

The animals were tethered in the center of the tent while the human volunteer slept on a cot protected by an untreated sand fly-proof mosquito net inside the tent trap. Baited tent trap and an un-baited control trap were arranged in a circular manner at a distance of about 30 meters from each other (Figure 1-C). The baits and un-baited traps rotated every night between different positions to eliminate location or site variations in sand fly abundance. Two replicate collections were performed on a total of 16 trapping nights. Some of the animals were provided with grass and straw to calm them down for the nightlong session. Collection of trapped sand flies from the interior walls and the roof of the tent traps was performed early in the mornings (06:30–08:00 hours) by three to four trained and experienced collectors (one person/net) using mouth aspirators while the animals were still inside the net to prevent sand flies from escaping. The collected sand flies were placed in small Barraud cages, each labeled corresponding to the host until processing in the field laboratory. Blood-fed females were separately sorted out and all sand fly specimens were preserved in 70% ethanol for later identification to species.

### Experiment II

#### Trapping small animals and evaluation of attractiveness to sand flies

Sherman-live traps and Tomahawk collapsible traps were used for trapping small animals needed for conducting host choice experiments. Traps baited with peanut butter were set near rock crevices, farm fields, rodent burrows and visible animal paths at night and at day time, and were checked for catches in the morning and in the evening, respectively. The only animals captured in this way were ground squirrel (*Xerus rutilus*), gerbil (*Tatera robusta*) and Cairo spiny mouse (*Acomys cahirinus*). Hares (*Lepus* sp.) were captured by chasing them from their hidings in the bush.

Square box traps (30cmx30cmx30cm) with entrance brass screen cones on the three sides were locally constructed based on the design of Turner and Hoogstraal [21], and baited with the above animals (each restrained in a cylindrical wire mesh cage) were used for the experiment. However, these traps failed to catch sand flies. Therefore, as an alternative approach, the host choice experiment was conducted by placing modified CDC traps in an up-draft position after removing the light bulbs. These were set up in caged animals and a blank cage as control (Figures 1 D-E). The unlit CDC traps were placed with their opening 5 cm above each animal. The respective sizes of the rectangular metal wire cages used for the hare and ground squirrel were 40 × 20 × 20 cm^3^ and 12 × 10 × 8 cm^3^, respectively. The smaller rodents were kept inside a cylindrical wire mesh cage (18 cm × 6 cm) with a metal lid at both ends. Like experiment one, caged animal baits and the blank control were placed in a circular manner at a distance of 30 m from each other. Experimental sessions started 1 h before sunset and terminated 1 h after sunrise, the following morning. The experiment was repeated 10 times totaling 10 trapping nights per bait and the animals were rotated between the different positions as above. In the mornings, sand fly specimens caught in the traps were collected using mouth aspirators, placed in separate Barraoud cages and transported to the field laboratory. Females were separated into fed and unfed, and preserved in 70% ethanol for later identification to species level.

### Sand fly identification

Collected sand fly specimens were mounted on microscope slides in Hoyer’s medium with their heads facing down and separated from thoraces and abdomens. The species were identified based on appropriate keys and other published materials [23-25].

### Ethical considerations

Informed consent was obtained from all human volunteers who participated in the host attractiveness experiments. Moreover, the experiments involving human volunteers and animals, described in this report, were ethically approved by the ethical review committee at the Medical Faculty, Addis Ababa University and the National Research Ethics Review Committee at the Ethiopian Ministry of Science and Technology. Moreover, fieldworks carried out in this study conformed to the International Guiding Principles for Biomedical Research Involving Animals developed by the Council for International Organizations of Medical Sciences and with the Standards for Human Care and Use of Laboratory Animals.

### Data analysis

Prior to data analysis, sand fly numbers were transformed to Log_10_ (x + 1) to fit normal distribution and control the variance. A univariate analysis of variance (ANOVA) was used to compare the mean number of *P. orientalis* attracted to different animal baits and control traps. Tukey’s Studentized test post hoc analysis was utilized to ascertain the extent of the difference between the groups in cases where ANOVA was significant. Probabilities of the F tests were at α = 0.05 level. The non-parametric equivalent test (Kruskal-Wallis test) was used when data did not conform to the normal distribution. For non-parametric comparisons, multiple-Mann–Whitney *U*-test was used and, *p*-values were adjusted with the Bonferroni correction to adjust for the inflation of type I errors when several Mann–Whitney tests were performed [26]. All statistical analyses were carried out using IBM SPSS statistics, version 19 for Windows (SPSS Inc., Chicago, IL, USA) and Microsoft® Office Excel 2007.

## Results

### Host attractiveness for *P. orientalis* and other sand flies

In experiment one involving domestic animals and human, a total of 21,144 sand flies belonging to six species of *Phlebotomus* and seven species of S*ergentomyia* were collected and identified (Table 1). Of these, 13,764 were males and 7,380 were females. The most abundant species was *P. orientalis* (54.36%) followed by *S. africana* (25.24%) of all collections.

**Table 1:** **Sand fly species captured in tent traps baited with different domestic animals or human volunteer in agricultural fields at Tahtay Adiyabo district**

	**Baits**								
**Sand fly species**	**Cow**	**Donkey**	**Human**	**Sheep**	**Goat**	**Dog**	**Chicken**	**Control**	**Total**
	**M/F**	**M/F**	**M/F**	**M/F**	**M/F**	**M/F**	**M/F**	**M/F**	
*P. orientalis*	5753/855	1661/1323	458/ 595	344/80	213/56	67/39	20/60	16/7	11,493
*P. rodhani*	0/0	0/0	0/ 0	0/0	4/0	0/0	0/0	0/0	4
*P. lesleyae*	1/4	1/4	0/9	0/2	5/5	0/1	0/2	1/1	36
*P. bergeroti*	0/0	0/0	0/0	0/0	4/0	0/0	0/0	0/0	4
*P. martini*	0/0	0/0	11/11	0/0	4/0	0/0	0/0	0/0	26
*P. heischi*	0/1	0/0	0/0	0/0	4/0	0/0	0/0	0/0	5
*S. africana*	305/198	278/203	190/258	568/286	507/257	599/250	553/155	650/79	5,336
*S. schwetzi*	367/526	433/756	112/105	204/181	109/290	64/173	21/37	7/30	3,415
*S. clydei*	27/97	23/114	4/5	12/76	23/90	14/45	1/1	3/3	538
*S. bedfordi group*	10/8	12/10	5/28	6/6	5/9	15/16	2/3)	1/0	146
*S. antennata group*	11/12	12/16)	7/6	14/9	4/9	5/15	6 (/2	5/5	137
*S. calcarata*	0/0	0/0)	0/0	0/0	0/0	0/1	0/0	0/0	1
*S. squamipleuris*	0/0	0/0	0/0	1/0	0/0	0/0	1/0	1/0	3
Total	6474/1701	2420/2426	787/1017	1149/640	882/716	764/540	604/216	684/24	21,144

M = Male; F = Female.

There were significant differences in the mean numbers of sand fly species caught by different baited traps (ANOVA, F _*(df =7)*_ = 67.93, *P* < 0.05, Table 2). All hosts, except chicken, attracted considerably more sand flies than the controls (Table 2). Increased attractions in decreasing order of magnitude were found in cow-baited, donkey-baited and human-baited tents. The cow- and donkey-baited tents had significantly higher attractions than all the other baits (*P* < 0.05).

**Table 2:** **Mean numbers (±SE) of sand fly specimens captured in tent traps baited with different domestic animals and human host in agricultural fields at Tahtay Adiyabo district**

**Bait types**	**Mean number ± SE of sand flies collected/ trap**
Cow	510.93 ± 75.87a
Donkey	302.94 ± 45.74b
Human	112. 81 ± 9.60c
Sheep	111.81 ± 20.94c
Goat	99.88 ± 11.52c
Dog	81.50 ± 20.15c
Chicken	51.25 ± 10.96d
Control	50.50 ± 8.61d

Mean values followed by the same letter on the same line are not significantly different (*P* < 0.05).

Animal baits differed substantially in their attractiveness to female and male *P. orientalis* (Table 3, Kruskal-Wallis test, *P* < 0.05). Cow-baited traps collected notably higher mean number of *P. orientalis* (mean = 510 flies/tent trap) than other baits and control traps. However, donkey-baited traps attracted the highest mean number of *P. orientalis* females (mean = 82.69) (Table 3) though it was not significantly different from cow (Multiple-Mann Whitney *U*-test, *P* > 0.01). Human bait was the third most effective attractant for collecting large numbers of *P. orientalis* females followed by sheep and goat, respectively. Dog and chicken-baited traps were the least attractive to *P. orientalis* females with no statistical difference between them and the control (Multiple-Mann Whitney *U*-test, *P* > 0.01) (Table 3).

**Table 3:** **Mean numbers (±SE) of female and male** ***P*** **.** ***orientalis*** **attracted to tent traps baited with different domestic animals and human host**

**Bait types**	**Mean number ± SE of sand flies collected/tent trap**	
	**Female**	**Male**
Cow	53.44 ± 3.19ab	359.56 ± 54.25a
Donkey	82.69 ± 10.81a	103.81 ± 21.16b
Human	37.19 ± 2.82c	28.63 ± 3.24c
Sheep	5.00 ± 1.12d	21.50 ± 4.79 cd
Goat	3.5 ± 1.19de	13.31 ± 3.34d
Dog	2.44 ± 0.99ef	4.19 ± 1.52e
Chicken	0.38 ± 0.25f	1.25 ± 0.61e
Control	0.44 ± 0.12f	1.00 ± 0.45e

Mean values followed by the same letter on the same line are not significantly different (*P* < 0.01; Multiple-Mann Whitney *U*-test).

**Figure 2:**
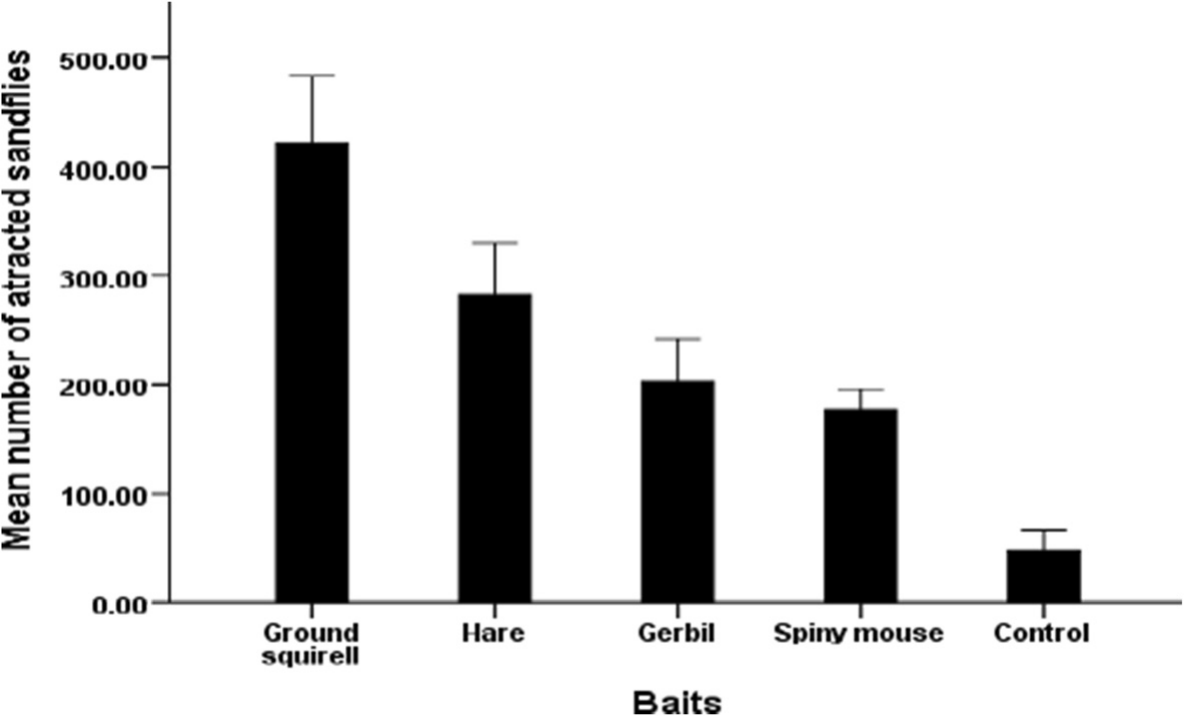
**Mean numbers (± standard errors) of sand fly specimens captured in traps baited with different small wild mammals at Tahtay Adiyabo district.**

In experiment two, comparing the attractiveness of small wild animals, 9,015 sand fly specimens (males: 3,831; females: 5,184) representing eleven species in two genera were captured (Table 4). As in experiment one, *P. orientalis* was the dominant species comprising 81.8% of the catch followed by *S. africana* (10.1%). There was a significant difference between the baits and the control (ANOVA, F _*(df =4)*_ = 23.16, *P* < 0.05; Figure 2). The mean number of sand flies attracted to ground squirrel was higher than that attracted to hare-baited traps, though these differences were not significantly different (*P* > 0.05). There was no statistically significant (*P* > 0.05) differential sand fly attraction between the spiny mouse (*A. cahirinus*) and gerbil (*T. robusta*).

**Table 4:** **Number of sand fly species attracted to different small wild mammals in agricultural fields at Tahtay Adiyabo district**

	**Baits**									
**Sand fly species**	**Squirrel**		**Hare**		**Gerbil**		**Spiny mouse**		**Control**	
	**M**	**F**	**M**	**F**	**M**	**F**	**M**	**F**	**M**	**F**
*P. orientalis*	955	2054	917	906	394	854	523	634	48	89
*P. rodhaini*	5	5	11	11	6	7	2	3	0	2
*P. lesleyae*	2	2	0	1	0	2	3	2	0	1
*P. martini*	1	0	0	0	0	0	0	0	0	0
*P. heischi*	1	2	3	2	0	0	1	1	0	0
*S. africana*	109	28	243	27	188	26	98	54	112	26
*S. schwetzi*	8	18	11	28	7	14	18	20	6	65
*S. clydei*	26	149	15	57	12	37	18	20	5	5
*S. bedfordi* group	6	1	6	0	11	0	3	3	4	2
*S. antennata* group	8	3	7	7	22	2	9	9	7	4
*S. adleri*	0	0	0	0	0	1	0	0	0	0
Total	1,121	2,262	1,213	1,039	640	943	675	746	182	194

M = Male; F = Female.

Analysis of variance (ANOVA) revealed that caged-animals differed in their attractiveness to both female and male *P. orientalis* (F _*(df =4)*_ =35.19; *P* < 0.05; Table 5). Higher mean numbers of *P. orientalis* females were attracted to ground squirrel than other baits and control traps (*P* < 0.05). The hare was the second attractive animal to *P. orientalis* females, followed by *T. robusta* and *A. cahirinus* with insignificant differences in their mean numbers.

**Table 5:** **Mean numbers (±SE) of female and male** ***P*** **.** ***orientalis*** **attracted to traps baited with different small wild mammals**

**Bait types**	**Mean number ± SE of sand flies captured/CDC trap**	
	**Female**	**Male**
Ground squirrel	256.75 ± 44.91a	119.38 ± 19.25a
Hare	113.25 ± 18.72b	114.63 ± 18.87ab
Spiny mouse	79.25 ± 14.05b	65.37 ± 7.77b
Gerbil	106.75 ± 28.26b	49.25 ± 11.75bc
Control	12.75 ± 2.91c	6.00 ± 1.53d

Mean values followed by the same letter on the same line are not significantly different (*P* < 0.05).

### Engorgement rates

In experiment one, of the total 6,239 host-seeking *P. orientalis* females trapped in baited-tent traps excluding human bait, 30.53% were freshly engorged. The mean numbers of blood engorged sand fly specimens differed among the other six hosts (Kruskal-Wallis test, *P* < 0.05, Table 6). *P. orientalis* fed most successfully on donkey (Mean = 78.56 engorged flies). Cow was the second preferred host. Conversely, this species fed less successfully on goat, sheep, dog and chicken in decreasing order with no significant difference (multiple-Mann Whitney *U*-test, *P* > 0.01).

**Table 6:** **Number and percentage of female sand fly species attracted and engorged on different domestic animals baits**

**Sand fly species**	**Percentage of blood fed females**					
	**Cow**	**Donkey**	**Sheep**	**Goat**	**Dog**	**Chicken**
	**% fed**	**% fed**	**% fed**	**% fed**	**% fed**	**% fed**
*P. orientalis*	72.8 (855)	92.6 (1323)	27.5 (80)	44.6 (56)	18 (39)	66.7 (6)
*P. lesleyae*	0 (4)	0 (4)	0 (2)	0 (5)	0 (1)	0 (2)
*P. heischi*	0 (1)	0 (0)	0 (0)	0 (0)	0 (0)	0 (0)
*S. africana*	0 (198)	0 (203)	0 (286)	0 (257)	0 (250)	0 (155)
*S. schwetzi*	1.9 (526)	0.7 (756)	0 (181)	0.7 (290)	0 (173)	0 (37)
*S. clydei*	3.1 (97)	0 (114)	0 (76)	2.2 (90)	0 (45)	0 (1)
*S. bedfordi group*	0 (8)	0 (10)	0 (6)	0 (9)	0 (16)	0 (13)
*S. antennata group*	0 (12)	0 (16)	0 (9)	0 (9)	0 (15)	0 (2)

Figures in brackets denote total number of sand fly females caught in various animal baited tent traps.

**Figure 3:**
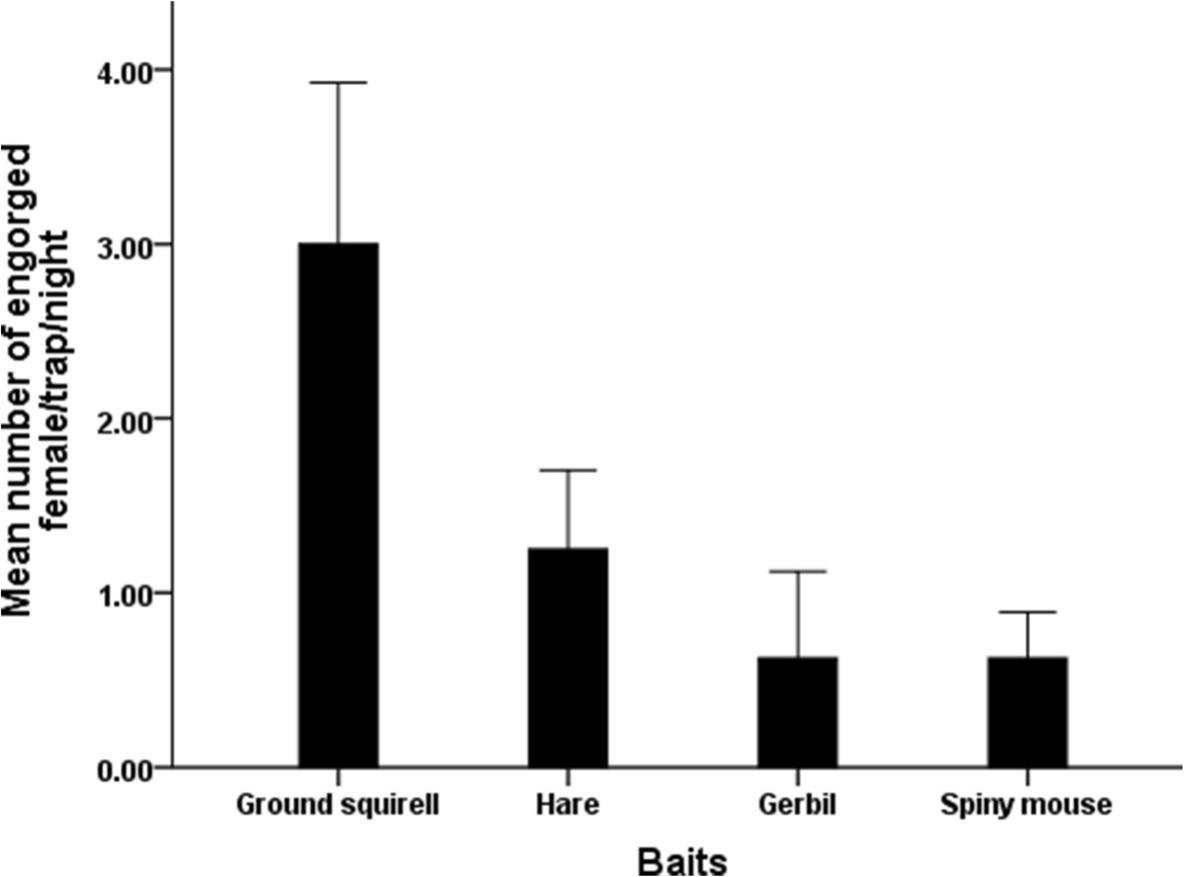
**Mean numbers (± standard errors) of engorged female** ***P. orientalis*** **on different species of wild small mammals.**

In experiment two involving small wild animals, only 1.08% (48/4,448 flies) were found with bloodmeals. Although the number of engorged *P. orientalis* was small, there were significant differences in the mean numbers of engorged females of sand fly specimens between the four bait species (ANOVA, F _(*df=3)*_ = 5.37; *P* = 0.005, Figure 3). Ground squirrel and hare were the preferred hosts over the two rodent species.

### Sex ratio

In experiment one**,** the total number of *P. orientalis* females caught by animal baited traps and control traps combined was smaller than that of males (7,380 female: 13,764 male). The overall female/male sex ratio for *P. orientalis* species was 0.54, which was significantly in favour of males (*P* < 0.05). It was only in human-baited traps that the female/male ratio (=1.3) was in favour of females (Table 7).

**Table 7:** **Sex ratio (F/M) of** ***P. orientalis*** **females and males attracted to traps baited with different animal and human hosts**

**Bait types**			
**Domestic animals**	F/M*	**Small wild animals**	F/M*
Cow	0.15	Ground squirrel	2.15
Donkey	0.8	Hare	0.9
Human	1.3	*Spiny mouse*	1.21
Sheep	0.23	*Gerbil*	2.17
Goat	0.26	Control	1.85
Dog	0.58		
Chicken	0.3		
Control	0.44		

*F/M = proportion of female: male *P. orientalis.*

In experiment two, the overall female/male sex ratio of *P. orientalis* attracted to host species and control traps was 1.6, which was significantly different (ANOVA, F _(*df=3)*_ = 3.66; *P* = 0.024), showing predominantly female sand fly attractiveness by all hosts except hare baited traps (Table 7).

## Discussion

Host preferences of vectors represent an important aspect of the bionomics of vector-borne disease dynamics, directly affecting the magnitude of disease transmission. The experiments described here demonstrated that *P. orientalis* females were attracted and engorged more frequently upon certain hosts than others. Host attractiveness to sand flies varies temporally and spatially; phenomena which could be associated with host body surface area, dose-specific responses to ubiquitous cues such as CO_2_ and host-specific odors [27-29].

In this experiment involving domestic animals and humans, higher numbers of *P. orientalis* females were attracted and engorged on donkey and cow than other hosts. Similar results have been previously recorded for Old as well as New World vectors [13,27,30]. These constitute favored bloodmeal sources for female *P. orientalis* as demonstrated in direct bloodmeal analysis by ELISA and PCR (Gebresilassie *et al.*, in preparation). The accessibility of bovine blood hosts to questing *P. orientalis* females in the peri-domestic habitats may provide zooprophylactic barrier potentially reducing human-vector contact, or it may aggravate the risk of VL infection. Studies in Nepal [31] showed that ownership or proximity of cattle was associated with significant protection of VL infection, whereas in India VL appeared to increase for those living in close proximity to cattle [32]. In Sudan, Mukhtar *et al.* [33] were able to detect the presence of anti-*Leishmania* antibodies in donkeys, cows, and goats. A recent study in Nepal also detected the presence of *Leishmania* DNA in domestic animals such as goats, cattle, and buffaloes, several months after the active transmission season [34]. Similarly, *L. donovani* DNA was detected in cattle, donkeys, sheep, and goats in cross-sectional studies in our study area (Rhoussova *et al.*, in preparation). Therefore, the role of cattle in the epidemiology of VL in our study area requires detailed and systematic investigation.

The current study as well as a previous study from Sudan has shown that humans are attractive hosts to *P. orientalis* [35]. Importantly, bloodmeal determination of engorged wild-caught females of *P. orientalis* in the same area revealed that 8.5% of females contain human blood origins (Gebresilassie *et al.*, in preparation). This finding supports the likelihood that *P. orientalis* is the vector of VL in these parts of East Africa since attraction to humans by a sand fly vector is a minimum requirement for disease transmission [36].

Relatively few *P. orientalis* females were attracted to and engorged upon sheep, goat, dog or chicken (Table 3). In agreement of this finding, bloodmeal analyses of engorged wild-caught females in the same area revealed that only a small proportion had fed upon these hosts ([13], Gebresilassie *et al.*, in preparation). Thus, our results do not support a role of goats, sheep, dogs and chickens as food source for *P. orientalis* as suggested in previous studies from Kenya [18,30] and Sudan [20] showing that these animals were highly attractive to *P. martini and P. orientalis*. These variations might be due to differences in the innate behavior of the sand fly species involved, and the experimental design used. Parasitological studies in Kenya also confirmed that sheep could not support the infection of *L. donovani* [37,38].

In the experiment using small mammals, *P. orientalis* was more attracted to ground squirrels (*X. rutilus*), followed by hares (*Lepus* sp.), gerbils *(T. robusta*) and spiny mice (*A. cahirinus*). However, the feeding rates in all cases were very low compared with baited-tent traps, probably because the attracted sand flies in this case were trapped before they had sufficient time to feed on the hosts. Rejection does not seem to be the case, since these small mammals are the common wild animals that *P. orientalis* would encounter in the wild including fissures in vertisols and fields, where humans and domestic animals are absent. It has previously been observed that wild-caught *P. orientalis* and *P. martini* had fed upon squirrels and rodents [14,39-41]. Different species of rodents have been identified as the reservoir hosts of *Leishmania* spp. in various parts of the world [11,42,43] and hares (*L. granatensis*) were recently incriminated as reservoir hosts of *L. infantum* in Spain [44,45]*.* The exact role of these animals in the epidemiology of VL in the study area remains to be explored.

Male sand flies predominated in the tent traps-baited with large domestic animals, indicating that mating occurs on the host. A swarming male population of *P. argentipes* and *Lu. longipalpis* were described close to animals used as bloodmeal sources by the females [46-48]. Unlike the larger domestic animals, however, the sex ratio in smaller wild animals was female biased except for hares. This higher proportion of female sand flies on smaller wild animals might be associated with the differences in body size of the animal baits or the design of trapping methods followed in both experiments.

## Conclusions

The tendency of female *P. orientalis* to engorge in large numbers on certain species of domestic as well as wild animals strongly indicated that the species is primarily zoophilic in its host preference with feeding habits that may vary depending on the availability of hosts. In addition, increased predilection of *P. orientalis* to bite cattle, the predominant domestic animal in our study area, may have a protective or increased exposure to VL, which requires further investigations. This zoophilic behavior can, however, be exploited for killing sand flies using pyrethroid insecticide treated animals [49]. Furthermore, detailed parasitological and xenodiagnostic studies, may shed some light on the epidemiology of kala-azar facilitating the implementation of effective control strategies.
